# Easily Conducted Tests During the First Week Post-stroke Can Aid the Prediction of Arm Functioning at 6 Months

**DOI:** 10.3389/fneur.2019.01371

**Published:** 2020-01-09

**Authors:** Emma Ghaziani, Christian Couppé, Volkert Siersma, Hanne Christensen, S. Peter Magnusson, Katharina S. Sunnerhagen, Hanna C. Persson, Margit Alt Murphy

**Affiliations:** ^1^Department of Physical and Occupational Therapy, Bispebjerg Hospital, Copenhagen, Denmark; ^2^Department of Orthopaedic Surgery M, Institute of Sports Medicine, Bispebjerg Hospital, Copenhagen, Denmark; ^3^Faculty of Health and Medical Sciences, Centre for Healthy Aging, University of Copenhagen, Copenhagen, Denmark; ^4^Research Unit for General Practice and Section of General Practice, Department of Public Health, University of Copenhagen, Copenhagen, Denmark; ^5^Department of Neurology, Bispebjerg Hospital, Copenhagen, Denmark; ^6^Faculty of Health and Medical Sciences, University of Copenhagen, Copenhagen, Denmark; ^7^Research Unit for Rehabilitation Medicine, Department of Clinical Neuroscience, Institute of Neuroscience and Physiology, University of Gothenburg, Sahlgrenska Academy, Gothenburg, Sweden

**Keywords:** acute stroke, arm paresis, arm recovery, arm functioning, prognostic models, prediction

## Abstract

**Background:** Prognostic models can estimate the recovery of arm functioning after stroke, guide the selection of individual training strategies, and inform patient selection in clinical trials. Several models for early prediction of arm recovery have been proposed, but their implementation has been hindered by insufficient external validation, limited evidence of their impact on patient outcomes, and reliance on predictors that are not feasible in regular clinical practice.

**Objectives:** To determine the predictive value of new and previously reported tests that can be easily conducted in regular clinical settings for early prognosis of two levels of favorable arm recovery at 6 months post-stroke.

**Methods:** We performed a secondary analysis of merged data (*n* = 223) from two Scandinavian prospective longitudinal cohorts. The candidate predictors were seven individual tests of motor function and the sensory function measured by the Fugl-Meyer Assessment of Upper Extremity within 7 days post-stroke, and the whole motor section of this assessment. For each candidate predictor, we calculated the adjusted odds ratio (OR) of two levels of residual motor impairment in the affected arm at 6 months post-stroke: moderate-to-mild (≥32 points on the motor section of the Fugl-Meyer Assessment of Upper Extremity, FMA-UE) and mild (FMA-UE ≥ 58 points).

**Results:** Patients with partial shoulder abduction (OR 14.6), elbow extension (OR 15.9), and finger extension (OR 9.5) were more likely to reach FMA-UE ≥ 32. Patients with full function on all individual motor tests (OR 5.5–35.3) or partial elbow extension, pronation/supination, wrist dorsiflexion and grasping ability (OR 2.1–18.3) were more likely to achieve FMA-UE ≥ 58 compared with those with absent function. Intact sensory function (OR 2.0–2.2) and moderate motor impairment on the FMA-UE (OR 7.5) were also associated with favorable outcome.

**Conclusions:** Easily conducted motor tests can be useful for early prediction of arm recovery. The added value of this study is the prediction of two levels of a favorable functional outcome from simple motor tests. This knowledge can be used in the development of prognostic models feasible in regular clinical settings, inform patient selection and stratification in future trials, and guide clinicians in the selection of individualized training strategies for improving arm functioning after stroke.

**Clinical Trial Registration:** ClinicalTrials.gov: NCT02250365, NCT01115348.

## Introduction

Stroke remains a major cause of disability worldwide (1). Arm paresis is one of the most common deficits after stroke and is present in 48–77% of patients at stroke onset (2–4). Arm paresis severely affects quality of life (5) and independence in activities of daily living (ADL) (6). Despite rehabilitation, only 12–34% of patients will achieve a complete functional recovery in terms of hand dexterity 6 months post-stroke (7, 8) at which time the restoration process seems to have reached a plateau (9, 10). Consequently, a large percentage of individuals with stroke will have to manage their daily lives with a non-functioning arm. Thus, recovery of the affected arm is a critical issue in stroke rehabilitation, particularly during the first 4–10 weeks when the restoration process is most pronounced (9–13).

A prognostic model is a formal combination of multiple predictors from which the risk of experiencing a specific endpoint within a specific period can be calculated for an individual patient with a given state of health (14). Prognostic models in stroke rehabilitation can assist clinicians in estimating the probability of an individual patient to achieve a favorable outcome over a specific period and guide the selection of the most appropriate intervention methods for the patient (14–16). However, prediction at individual level is still a challenge. Firstly, it is not advised to implement prognostic models in clinical practice before undergoing a rigorous development process, including validation outside the specific context in which the models initially were developed, and evaluation of their impact on clinical decision making and patient outcomes (14, 15). Several models for early prediction of arm recovery have been proposed (7, 8, 16–23), but their widespread implementation in clinical practice has been hindered by insufficient external validation and limited evidence of the impact on patient outcomes; few studies have been conducted on these areas (24, 25). Secondly, prognostic models including direct measurement of the corticospinal tract's integrity are dependent on expensive equipment (transcranial magnetic stimulation, magnetic resonance imaging), and specialist expertise (16, 17). Currently, neurophysiological assessments are the only available tools to provide early discrimination between favorable and unfavorable outcomes in patients with initially very severe motor impairment (16, 17). Unfortunately, these assessments are often not available in regular clinical practice for purposes of prediction of arm recovery.

A broad range of arm tests that clinicians can perform bedside as part of the initial stroke assessment without special equipment have shown to predict the affected arm functioning at later stages. Some active finger extension measured on day 7 after stroke has been reported to predict a better arm recovery throughout the first 6 months post-stroke (26). The early ability to perform proximal arm movement (shoulder shrug, shoulder abduction) was likewise linked to more favorable hand dexterity at 1–3 months after stroke (27). The initial severity of arm paresis has been consistently found to predict the actual level of arm functioning measured up to 12 months post-stroke (7, 23, 28–34) and the amount of improvement taking place over this period (20, 21, 24). Different combinations of tests assessing proximal (shoulder abduction, elbow flexion, placing the hand on the top of the head) and distal (finger extension, grip strength) function in the affected arm early post-stroke have also been suggested as multivariable prognostic models (8, 16–18, 22, 23). Furthermore, the presence of sensory impairments during the first weeks after stroke has indicated a less favorable outcome in terms of hand dexterity (19, 29, 32). The need for external validation of already proposed predictors, as well as for development and validation of additional predictors accessible for clinical practice remains (34–36). Therefore, the objective of this study was to determine the predictive value of new and previously reported tests that can be easily conducted in regular clinical settings for early prognosis of two levels of favorable arm recovery at 6 months post-stroke.

## Materials and Methods

The current study is reported according to the STROBE (Strengthening the Reporting of Observational Studies in Epidemiology) guidelines (37).

### Study Design

This study was a secondary analysis of merged data from two independent studies and was initiated as a collaboration between two research groups. The first study was a prospective longitudinal cohort study, the Stroke Arm Longitudinal Study at University of Gothenburg (SALGOT), Sweden, aiming to describe the recovery of arm functioning during the first 12 months after stroke (38). All included patients received standardized rehabilitation according to the Swedish National Stroke Guidelines (39) and did not participate in any other intervention studies. The second was a randomized controlled trial conducted in the stroke rehabilitation unit of Bispebjerg and Frederiksberg Hospital, Copenhagen, Denmark, and examined the effect of electrical somatosensory stimulation (ESS) delivered prior to task-oriented arm training during early inpatient rehabilitation on the recovery of arm functioning at 6 months post-stroke (the ESS trial) (40, 41). The ESS trial demonstrated no difference between the intervention and the control group (42), making it possible to merge the two cohorts for the purpose of the present study. Detailed information on the type and amount of delivered rehabilitation in the ESS trial is available elsewhere (42).

### Trial Settings and Participants

In the SALGOT-study (38), all patients consecutively admitted to the largest of the three stroke units of the Sahlgrenska University Hospital, Gothenburg, were screened from February 2009 to December 2010 (except a total of a 145-day period due to administrative reasons) using following inclusion criteria: (a) first-ever stroke (ischaemic or haemorrhagic stroke); (b) age ≥ 18 years; (c) impaired arm function measured on day 3 (±1 day) after stroke (<66 points on the motor section the Fugl-Meyer Assessment of Upper Extremity); (d) admission to the stroke unit ≤3 days after stroke onset; and (e) residence in the Gothenburg urban area. Patients were excluded if one of the following criteria was present: (a) injury/condition prior to the stroke that limited the use of the affected arm; (b) severe multi-impairment or diminished physical condition prior to stroke; (c) short life expectancy; and (d) not able to communicate in Swedish.

In the ESS-trial, all patients consecutively admitted to the stroke rehabilitation unit of Bispebjerg and Frederiksberg Hospital from October 2014 to March 2017 (except a total of a 6-month period due to holidays and recruitment/training of new trial staff) were assessed for eligibility according to following criteria: (a) acute ischaemic or haemorrhagic stroke; (b) age ≥18 years; (c) impaired arm function (<66 points the Fugl-Meyer Assessment of Upper Extremity) measured within the first week post-stroke; (d) residence in the hospital's catchment area for stroke rehabilitation; (e) no severe pre-stroke disability (modified Rankin Scale < 5 points); (f) no contraindications to ESS (pacemaker, skin impairment); (g) possibility for initiating the ESS-intervention within 7 days post-stroke due to medical or logistical issues; (h) no cognitive dysfunctions or poor communication skills in Danish that impeded the ability to provide informed consent; (i) complete recovery of the affected arm from a previous stroke; and (j) no participation in other biomedical intervention trials within the last 3 months.

### Outcome

The outcomes to be predicted were a moderate-to-mild (FMA-UE ≥ 32) and a mild (FMA-UE ≥ 58) residual motor impairment in the affected arm measured by the motor section of the Fugl-Meyer Assessment of Upper Extremity (43) (referred to as FMA-UE in the remaining article) at 6 months post-stroke. Previous research has shown that patients with moderate motor impairment (FMA-UE ≥ 32) (44) were able to perform at least basic ADL such as drinking from a glass with their affected arm (18, 45). A minimum of 58 points on FMA-UE has been suggested to represent the lower limit of a mild impairment level (44), indicating a high probability that the affected arm will routinely be used in ADL (46), i.e., an almost complete arm recovery.

FMA-UE has an excellent validity, and inter- and intra-observer reproducibility (43, 47, 48). A maximum score of 66 points indicates normal arm function. Among limitations, FMA-UE has shown to have a ceiling effect (48, 49), requires training to administrate, and it is considered time-consuming (30 min) for regular use in acute clinical settings (50).

### Candidate Predictors

The selected candidate predictors were easily conducted tests (i.e., time efficient and easy to perform) of proximal and distal motor function, and sensory function. Specifically, we examined the predictive value of seven individual items from the FMA-UE assessed during the first week post-stroke (baseline): (i) shoulder abduction within synergies (subscale A.II), (ii) elbow extension within synergies (subscale A.II), (iii) forearm pronation/supination with 90-degree elbow flexion (subscale A.III), (iv) wrist stability at 15-degree dorsiflexion with 90-degree elbow flexion (subscale B), (v) finger mass extension (subscale C), (vi) pincer grasp (subscale C), and (vii) cylinder grasp (subscale C). Each motor item is scored on a 3-level ordinal scale (0: absent, 1: partial, 2: full movement) based on the clinician's observation of the patient's performance. We also examined the predictive value of the sensory function measured with the sensory section of the Fugl-Meyer Assessment of Upper Extremity (43) and dichotomized into intact sensory function (12 out of 12 points) and sensory impairment (<12 points). Finally, we examined the predictive property of the entire FMA-UE measured at baseline. The same established cut-offs indicating severe (FMA-UE 0-31), moderate (FMA-UE 32-57), and mild (FMA-UE 58-66) motor impairment were used. Detailed instructions for conducting the FMA-UE are available elsewhere (51).

### Potential Confounders

The potential confounders considered in this study were: age, sex, living alone/with others, previous stroke, type of stroke, affected dominant hand, leg paresis, aphasia, stroke severity (Scandinavian Stroke Scale, SSS) (52), pre-stroke physical inactivity (Saltin-Grimby Physical Activity Level Scale, SGPALS-4) (53), number of hospital days, number of days from stroke onset until the assessment of candidate predictors, and sample (ESS/SALGOT) to adjust for design differences in the two studies. The SALGOT-study assessed the stroke severity using the National Institutes of Health Stroke Scale (NIHSS-scale) (54), and the pre-stroke physical activity level with SGPALS-6 (55). For this study, the NIHSS-values were converted into SSS-values using the mathematical equation SSS = 50.37–1.63^*^NIHSS (56); categories 1 and 2 of SGPALS-6 were merged into category 1 of GSPALS-4, and categories 5 and 6 of SGPALS-6 were merged into category 4 of SGPALS-4.

### Sample Size

**Figure 1:**
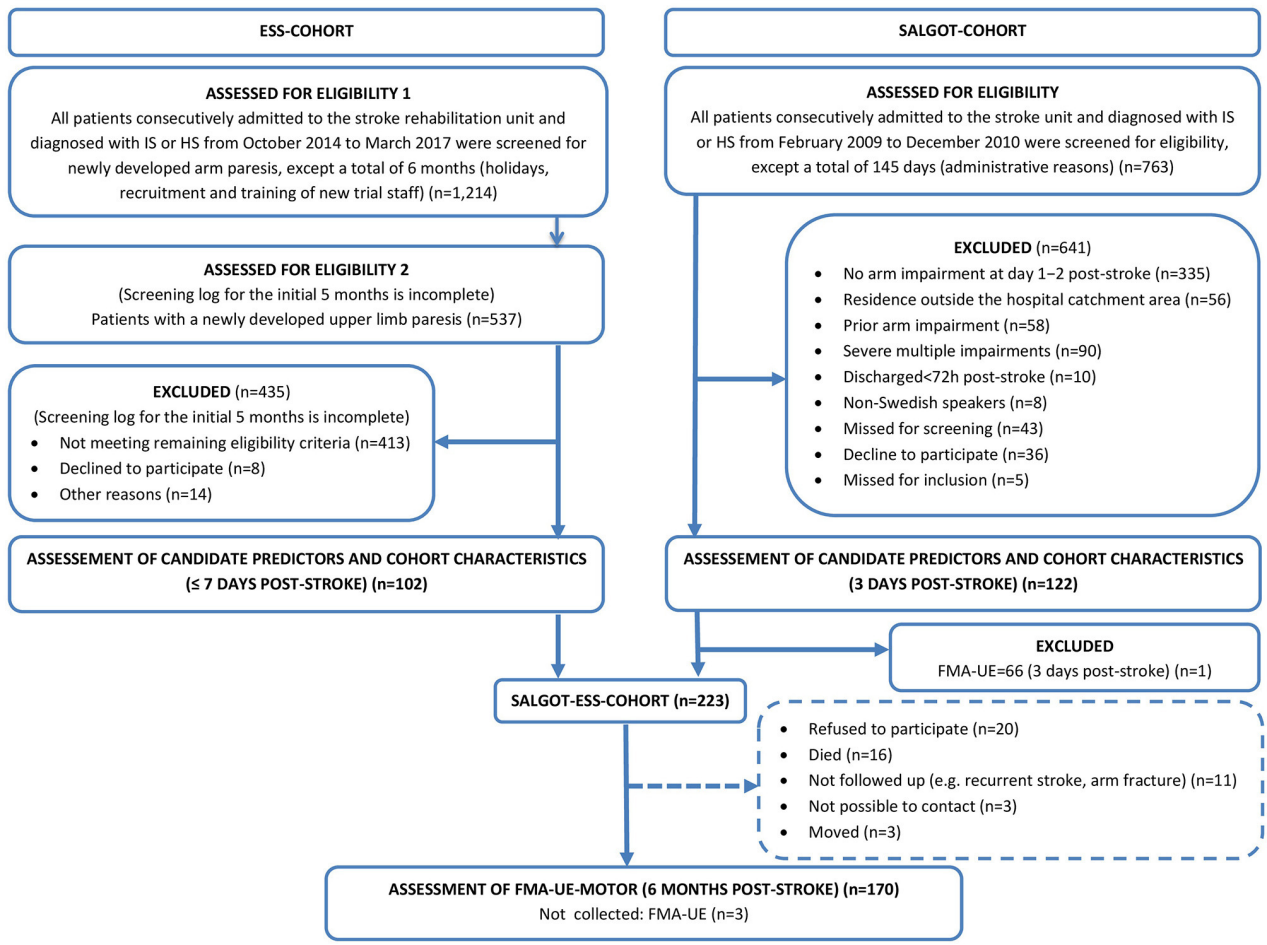
Flowchart of the inclusion process. ESS, the Electrical Somatosensory Stimulation trial; SALGOT, the Stroke Arm Longitudinal Study at the University of Gothenburg; FMA-UE, the motor section of the Fugl-Meyer Assessment of Upper Extremity (0–66 points).

The cohort was generated by merging individual participant data from the SALGOT-study (*n* = 121) and the ESS-trial (*n* = 102). For further details, see Figure 1. As suggested by the literature on prognostic research methods (15), with a total sample size of 223 participants, there are at least 10 observations for each of the eight candidate predictors (see section Candidate Predictors) and each of the 13 potential confounders (see section Missing Data).

### Statistical Methods

The data in this study was originally collected for the purpose of a clinical trial and an observational study. The statistical analyses we performed on the merged data were adjusted for the data source (variable: sample).

Descriptive statistics were used for presenting various baseline characteristics (e.g., demographics, clinical characteristics, risk factors) of the entire SALGOT-ESS-cohort, and of the subgroups emerged when stratifying the cohort by the 6-month outcome level (FMA-UE < 32/≥32, FMA-UE < 58/≥58). We employed the Wilcoxon Non-Parametric Test and Fisher's Exact Test to examine statistical differences on baseline characteristics between the subgroups.

For each candidate predictor we used logistic regression to calculate the odds ratio (OR) of a favorable outcome (FMA-UE ≥ 32 and ≥58) among patients with partial or full motor function (item score: 1 or 2), and intact sensory function (FMA-UE-sensory = 12) in comparison with the group of patients with absent motor function (item score: 0) and sensory impairments (<12 points on the sensory function of the Fugl-Meyer Assessment of Upper Extremity). Three logistic regression analyses, adjusting for an increasing number of confounders, were performed for each candidate predictor: (a) unadjusted (ORu); (b) partially adjusted (ORp) for: sample, sex, age, and living arrangement, and (c) fully adjusted (ORf) for: sample, age, sex, living arrangement, previous stroke, type of stroke, affected dominant hand, leg paresis, aphasia, stroke severity, pre-stroke physical inactivity, number of hospital days, and number of days from stroke onset until the assessment of candidate predictors. Analyses were performed with SAS version 9.4. The statistical significance was set to 1% to guard against false detection due to multiple comparisons.

### Missing Data

Missing outcome data could well be related to the patients' condition and will cause bias if naively omitted. We adjusted for differential dropout by weighting the non-missing observations with the inverse of the probability of this value being observed (57). These probabilities were for each observation estimated from a logistic regression model on the observation being missing including the same covariates as the fully adjusted model c) (see section Statistical Methods).

## Results

Figure 1 presents the flowchart of the participant inclusion process. The merged SALGOT-ESS-cohort comprised 223 study participants. Candidate predictors, demographics, risk factors and clinical characteristics of the cohort were recorded on day 3 (median) (Figure 1, Table 1). Complete data on the predicted outcomes at 6 months post-stroke were available for 167 out of the 223 participants (Figure 1, Table 1).

**Table 1:** Characteristics of the SALGOT-ESS-cohort.

	**Entire cohort**	**ESS cohort**	**SALGOT cohort**	***p*-value**	**Subgroups according to the FMA-UE at 6 months post-stroke (*****n*** **=** **167)**					
	**(*n* = 223)**	**(*n* = 102)**	**(*n* = 121)**		**<32**	**≥32**	***p*-value**	**<58**	**≥58**	***p*-value**
**Demographic characteristics at baseline**										
Age, years, median (Q1–Q3) (min–max)	71 (63–80) (26–95)				71 (63–78) (55–90)	69 (62–80) (34–92)	0.750	73 (63–80) (34–92)	68 (62–77) (38–91)	0.154
Sex, *n* (%)										
Men	120 (54)				24 (65)	68 (52)	0.086	38 (55)	54 (57)	0.532
Women	103 (46)				11 (35)	64 (48)		35 (48)	40 (43)	
Living arrangement, *n* (%)										
Living alone	112 (50)				17 (49)	68 (52)	0.849	35 (48)	50 (53)	0.535
Living with others	111 (50)				18 (51)	64 (48)		38 (52)	44 (47)	
**Risk factors for stroke at baseline**										
Previous stroke, yes, *n* (%)	22 (10)				4 (11)	12 (9)	0.746	9 (12)	7 (7)	0.303
Previous transient ischaemic attack, yes, *n* (%)	2 (1)				0 (0)	2 (2)	1.000	1 (1)	1 (1)	1.000
Previous atrial fibrillation, yes, *n* (%)	45 (20)				6 (17)	22 (17)	1.000	13 (18)	15 (16)	0.835
Previous myocardial infarction, yes, *n* (%)	8 (4)				2 (5)	4 (3)	0.606	2 (3)	4 (4)	0.697
Previous angina pectoris, yes, *n* (%)	4 (2)				2 (6)	2 (2)	0.193	3 (4)	1 (1)	0.319
Diabetes, yes, *n* (%)	28 (13)				3 (9)	13 (10)	1.000	8 (11)	8 (9)	0.607
Psychiatric disorder, yes, *n* (%)	10 (4)				1 (3)	5 (4)	1.000	2 (3)	4 (4)	0.697
Heart failure, yes, *n* (%)	21 (9)				3 (9)	11 (8)	1.000	7 (10)	7 (7)	0.779
Hypertension, yes, *n* (%)	120 (54)				21 (60)	68 (52)	0.447	45 (62)	44 (47)	0.062
Peripheral arterial disease, yes, *n* (%)	3 (1)				1 (3)	2 (2)	0.508	2 (3)	1 (1)	0.581
Hyperlipidaemia, yes, *n* (%)	37 (17)				4 (11)	28 (21)	0.233	11 (15)	21 (22)	0.321
Other diseases, yes, *n* (%)	79 (35)				9 (26)	44 (33)	0.422	27 (37)	26 (28)	0.241
Overweight (BMI ≥ 25), *n* (%)	93 (42)				15 (43)	66 (50)	0.567	36 (49)	45 (48)	0.877
Physically inactive pre-stroke (SGPALS = 1), *n* (%)	53 (25)				5 (15)	31 (24)	0.543	20 (29)	16 (18)	0.221
**Clinical characteristics at baseline**										
Type of stroke, *n* (%)										
Haemorrhagic stroke	41 (18)				7 (20)	29 (22)	1.000	19 (26)	17 (18)	0.256
Ischaemic stroke	182 (82)				28 (80)	103 (78)		54 (74)	77 (82)	
TOAST classification of subtypes of ischemic stroke, *n* (%)										
Large-artery atherosclerosis	28 (15)				4 (14)	18 (17)	0.566	9 (17)	13 (17)	0.765
Cardioembolism	51 (28)				10 (36)	23 (22)		15 (28)	18 (23)	
Small-artery occlusion	78 (43)				10 (36)	50 (49)		26 (48)	34 (44)	
Stroke of other determined etiology	10 (6)				2 (7)	6 (6)		2 (4)	6 (8)	
Stroke of undetermined etiology	15 (8)				2 (7)	6 (6)		2 (4)	6 (8)	
Acute treatment, yes, *n* (%)										
Thrombolysis	30 (13)				4 (11)	17 (13)	1.000	9 (12)	12 (13)	1.000
Thrombectomy	6 (3)				1 (3)	3 (2)	1.000	2 (3)	2 (2)	1.000
Stroke severity (SSS), *n* (%)										
Major stroke (SSS ≤ 25)	29 (13)				**10 (29)**	**7 (5)**	**≤0.01**	12 (16)	5 (5)	0.021
Moderate-to-mild stroke (SSS > 25)	194 (87)				25 (71)	125 (95)		61 (84)	89 (95)	
FMA-UE at baseline, median (Q1–Q3) (min–max)	36 (8–55) (0–65)	37 (11–51) (0–62)	21 (4–56) (0–65)	0.185	**4 (3–8) (0–13)**	**47 (21–56) (0–65)**	**≤0.01**	**8 (4–19) (0–57)**	**51 (41–58) (4–65)**	**≤0.01**
Affected arm, right, *n* (%)	108 (48)				13 (37)	62 (47)	0.342	30 (41)	45 (48)	0.434
Dominant hand, right, *n* (%)	217 (97)				35 (100)	127 (96)	0.585	71 (97)	91 (97)	1.000
Affected dominant hand, yes, *n* (%)	106 (48)				13 (37)	61 (46)	0.444	28 (38)	46 (49)	0.209
Aphasia, yes, *n* (%)	49 (22)				10 (29)	24 (18)	0.236	19 (26)	15 (16)	0.123
Leg paresis, yes, *n* (%)	139 (63)				**32 (86)**	**73 (56)**	**≤0.01**	**59 (81)**	**44 (47)**	**≤0.01**
FMA-UE at 6 months, median (Q1–Q3) (min–max)	60 (41–64) (4–66)	58 (49–64) (9–66)	60 (23–65) (4–66)	0.836						
Change in FMA-UE from baseline to 6 months, median (Q1–Q3) (min–max)	13 (6–24) (−2–59)	14 (7–25) (−2–53)	13 (6–24) (−2–59)	0.654						
No. of hospital days, median (Q1–Q3) (min–max)	21 (13–34) (2–100)				**37 (22–45) (10–100)**	**17 (12–30) (2–59)**	**≤0.01**	**31 (18–43) (8–100)**	**16 (9–26) (2–56)**	**≤0.01**
No. of days from stroke onset to the measurement of candidate predictors (baseline), median (Q1–Q3) (min–max)	3 (3–5) (0–7)				3 (3–5) (0–7)	3 (3–5) (2–7)	0.228	3 (3–6) (0–7)	3 (3–4) (2–7)	0.117

*BMI, Body Mass Index; SGPALS, Saltin-Grimby Physical Activity Level Scale; SSS, Scandinavian stroke scale; FMA-UE, the motor section of the Fugl-Meyer Assessment of Upper Extremity (0–66 points). P ≤ 0.01 are in boldface*.

The characteristics of the entire SALGOT-ESS-cohort and of the 6-month outcome subgroups are shown in Table 1. At baseline, the median age was 71 years, and the proportion of men was 54%. Half of the participants were living alone. The most prevalent risk factors for stroke were: (a) hypertension: 54%, (b) overweight (Body Mass Index ≥ 25): 42%, (c) other diseases: 35%, and (d) pre-stroke physical inactivity: 25%. Most participants had an ischaemic stroke (82%) due to small-artery occlusion (43%) and suffered a moderate-to-mild stroke (87%). The arm function was moderately impaired (FMA-UE = 36, median) with no statistically significant difference between the ESS- and the SALGOT-cohort; 63% had leg paresis and 22% aphasia. The median number of hospital days was 21. The subgroups with a less favorable 6-month outcome (FMA-UE < 32 and <58) comprised a significantly higher percentage of participants with major strokes, leg paresis, more impaired arm motor function at baseline, and a longer hospital stay. On average, study participants improved 13 points (median) on the FMA-UE during the course of the study, reaching a mild residual motor impairment (FMA-UE: 60 points, median) at 6 months post-stroke. There were no differences in terms of 6-month FMA-UE and change in FMA-UE between the ESS- and the SALGOT-cohort.

Tables 2, 3 show the main results of this study. Overall, the odds ratios (OR) of a favorable outcome (FMA-UE ≥ 32 and ≥58) were significantly higher among participants with partial/full motor function and intact sensory function compared with patients with absent motor function and sensory impairment at baseline. Since the estimated predictive values (OR) were less accurate due to large 95% confidence intervals (95% CI), the lower limits of the 95% CI were used as conservative estimates. Thus, when adjusting for all confounders (ORf), the probability of achieving a moderate-to-mild level of residual motor impairment (FMA-UE ≥ 32) in the affected arm at 6 months post-stroke was at least 14.6 times higher in participants who were able to abduct the shoulder partially within synergies at baseline compared with those patients who did not show any active movement. The fully adjusted predictive values for the remaining motor candidate predictors of FMA-UE ≥ 32 were not possible to quantify (it is estimated as infinity) (Table 2). However, in the partially adjusted analyses, partial elbow extension within synergies (ORp: at least 15.9) and finger mass extension (ORp: at least 9.5) at baseline were significantly associated with FMA-UE ≥ 32 at 6 months.

**Table 2:** Odds ratio of FMA-UE≥ 32 at 6 months post-stroke for each candidate predictor.

**≤7 days post-stroke**	**6 months post-stroke**		**Predictive value**								
**Candidate predictors**	**No. of patients with FMA-UE**		**Unadjusted analyses**			**Partially adjusted analyses**			**Fully adjusted analyses**		
	**<32**	**≥32**	**ORu**	**95% CI**	***p***	**ORp**	**95% CI**	***p***	**ORf**	**95% CI**	***p***
**PROXIMAL ARM FUNCTION**											
**Shoulder abduction**											
0: Absent = Reference	33	22									
1: Partial	2	35	**27.2**	**(5.9; 126.5)**	**≤0.01**	**43.9**	**(8.8; 219)**	**≤0.01**	**129.7**	**(14.6; 1,153.1)**	**≤0.01**
2: Full	0	74	∞			∞			∞		
**Elbow extension**											
0: Absent = Reference	34	22									
1: Partial	1	31	**61.9**	**(7.9; 488.3)**	**≤0.01**	**108.3**	**(15.9; 739.7)**	**≤0.01**	∞		
2: Full	0	78	∞			∞			∞		
**DISTAL ARM FUNCTION**											
**Pronation/supination**											
0: Absent = Reference	35	30									
1: Partial	0	40	∞			∞			∞		
2: Full	0	61	∞			∞			∞		
**Wrist dorsiflexion**											
0: Absent = Reference	35	35									
1: Partial	0	46	∞			∞			∞		
2: Full	0	50	∞			∞			∞		
**Finger extension**											
0: Absent = Reference	34	24									
1: Partial	1	35	**59.6**	**(7.6; 466. 8)**	**≤0.01**	**70.2**	**(9.5; 516.8)**	**≤0.01**	∞		
2: Full	0	73	∞			∞			∞		
**Pincer grasp**											
0: Cannot grasp = Reference	35	48									
1: Can grasp, but not hold against a tug	0	31	∞			∞			∞		
2: Can hold against a tug	0	52	∞			∞			∞		
**Cylinder grasp**											
0: Cannot grasp = Reference	35	46									
1: Can grasp, but not hold against a tug	0	28	∞			∞			∞		
2: Can hold against a tug	0	57	∞			∞			∞		
**SENSORY FUNCTION**											
Sensory impairment = Reference	28	56									
Intact sensory function	7	73	**6**	**(2.4; 14.9)**	**≤0.01**	**8.3**	**(2.7; 25.4)**	**≤0.01**	**8.3**	**(2; 33.9)**	**≤0.01**
**FMA-UE**											
Severe impairment = Reference	35	39									
Moderate	0	64	**∞**			**∞**			**∞**		
Mild impairment	0	28	**∞**			**∞**			**∞**		

*95% CI, 95% confidence interval; FMA-UE, the motor section of the Fugl-Meyer Assessment of Upper Extremity (0–66 points); ORu, odds ratio, unadjusted; ORp, odds ratio adjusted for sample, sex, age, living arrangement; ORf, odds ratio adjusted for sample, age, sex, living arrangement, previous stroke, type of stroke, affected dominant hand, leg paresis, aphasia, stroke severity, pre-stroke physical activity level, number of hospital days, and number of days from stroke onset to the assessment of candidate predictors. P ≤ 0.01 are in boldface*.

**Table 3:** Odds ratio of FMA-UE ≥ 58 at 6 months post-stroke for each candidate predictor.

**≤7 days post-stroke**	**6 months post-stroke**		**PREDICTIVE VALUE**								
**Candidate predictors**	**No of patients with FMA-UE**		**Unadjusted model**			**Partially adjusted model**			**Fully adjusted model**		
	**<58**	**≥58**	**ORu**	**95% CI**	***p***	**ORp**	**95% CI**	***p***	**ORf**	**95% CI**	***p***
**PROXIMAL ARM FUNCTION**											
**Shoulder abduction**											
0: Absent = Reference	45	10									
1: Partial	17	20	**5.6**	**(2.2; 14.6)**	**≤0.01**	**6.5**	**(2.3; 18.5)**	**≤0.01**	3.8	(1.2; 12.4)	0.03
2: Full	10	64	**26.5**	**(9.9; 70.6)**	**≤0.01**	**33.1**	**(11.5; 90)**	**≤0.01**	**21.3**	**(5.5; 83.2)**	**≤0.01**
**Elbow extension**											
0: Absent = Reference	50	6									
1: Partial	14	18	**10.1**	**(3.3; 31)**	**≤0.01**	**13.2**	**(3.9; 45.3)**	**≤0.01**	**12.1**	**(2.1; 69.1)**	**≤0.01**
2: Full	8	70	**77.1**	**(24.7; 240.3)**	**≤0.01**	**118.6**	**(31; 453.4)**	**≤0.01**	**532.4**	**(35.1; 8,087)**	**≤0.01**
**DISTAL ARM FUNCTION**											
**Pronation/supination**											
0: Absent = Reference	57	8									
1: Partial	9	31	**24.5**	**(8.5; 70.9)**	**≤0.01**	**41.1**	**(11; 153.1)**	**≤0.01**	**55.9**	**(8.7; 360.4)**	**≤0.01**
2: Full	6	55	**71.8**	**(23.1; 222.7)**	**≤0.01**	**166,6**	**(44.7; 620.6)**	**≤0.01**	**377,4**	**(35.3; 4,037.5)**	**≤0.01**
**Wrist dorsiflexion**											
0: Absent = Reference	61	9									
1: Partial	8	38	**31.9**	**(11.2; 91.2)**	**≤0.01**	**40.9**	**(12.8; 131.6)**	**≤0.01**	**118.1**	**(18.3; 762)**	**≤0.01**
2: Full	3	47	**111.8**	**(28.4; 440.4)**	**≤0.01**	**118.1**	**(26.4; 528.1)**	**≤0.01**	**248.5**	**(24.9; 2,481.2)**	**≤0.01**
**Finger extension**											
0: Absent = Reference	52	6									
1: Partial	14	22	**13,1**	**(4.3; 39.5)**	**≤0.01**	**19.2**	**(5.7; 64.1)**	**≤0.01**	8.2	(1.4; 47.6)	0.02
2: Full	7	66	**74**	**(23; 238.8)**	**≤0.01**	**97.9**	**(27.6; 346.5)**	**≤0.01**	**113.2**	**(18.3; 702.5)**	**≤0.01**
**Pincer grasp**											
0: Cannot grasp = Reference	62	21									
1: Can grasp, but not hold against a tug	7	24	**10.3**	**(3.8; 27.6)**	**≤0.01**	**11.9**	**(4.2; 33.6)**	**≤0.01**	**14.6**	**(3.2; 66.1)**	**≤0.01**
2: Can hold against a tug	3	49	**46.3**	**(13; 165.2)**	**≤0.01**	**51.8**	**(15; 191)**	**≤0.01**	**88.4**	**(9.5; 819.7)**	**≤0.01**
**Cylinder grasp**											
0: Cannot grasp = Reference	62	19									
1: Can grasp, but not hold against a tug	5	23	**16.8**	**(5.6; 50.6)**	**≤0.01**	**21.8**	**(6.3; 74.7)**	**≤0.01**	**37.4**	**(5; 279)**	**≤0.01**
2: Can hold against a tug	5	52	**35.3**	**(12.2; 102)**	**≤0.01**	**40.3**	**(13.7; 184.7)**	**≤0.01**	**70.7**	**(10.8; 463.7)**	**≤0.01**
**SENSORY FUNCTION**											
Sensory impairment = Reference	53	31									
Intact sensory function	17	63	**6.8**	**(3.4; 13.7)**	**≤0.01**	**6.4**	**(3.1; 13.3)**	**≤0.01**	**5.8**	**(2.2; 15.8)**	**≤0.01**
**FMA-UE**											
Severe impairment = Reference	61	13									
Moderate impairment	11	53	**24**	**(9.8; 58.6)**	**≤0.01**	**29.9**	**(11.3; 78.9)**	**≤0.01**	**24.6**	**(7.5; 81,2)**	**≤0.01**
Mild impairment	0	28	**∞**			**∞**			**∞**		

*95% CI, 95% confidence interval; FMA-UE, the motor section of the Fugl-Meyer Assessment of Upper Extremity (0–66 points); ORu, odds ratio, unadjusted; ORp, odds ratio adjusted for sample, sex, age, living arrangement; ORf, odds ratio adjusted for sample, age, sex, living arrangement, previous stroke, type of stroke, affected dominant hand, leg paresis, aphasia, stroke severity, pre-stroke physical activity level, number of hospital days, and number of days from stroke onset to the assessment of candidate predictors. P ≤ 0.01 are in boldface*.

The highest odds of achieving a mild level of residual motor impairment (FMA-UE ≥ 58) at 6 months were observed for patients with full pronation/supination with 90-degree elbow flexion (ORf: at least 35.3), full elbow extension within synergies (ORf: at least 35.1), full or partial wrist dorsiflexion (ORf: at least 24.9 or 18.3, respectively), and full finger mass extension (ORf: at least 18.3) at baseline (Table 3). Patients demonstrating full shoulder abduction within synergies, partial elbow extension within synergies, partial pronation/supination with 90-degree elbow flexion, and ability to hold a pen or cylinder against gravity or resistance had more modest, but still significantly higher chances of achieving FMA-UE ≥ 58 at 6 months after stroke (ORf: at least 2.1–10.8) (Table 3).

Patients with intact sensory function at baseline were at least two times more likely to achieve a moderate-to-mild level of motor impairment at 6 months post-stroke (Table 2) and at least 2.2 times more prone to achieve an almost complete arm recovery (Table 3) compared with patients with sensory impairment.

Having a moderate motor impairment at baseline improved the chances of achieving a mild residual impairment at 6 months by at least 7.5 times (Table 3). The remaining OR were estimated as infinity.

## Discussion

This study evaluated the value of individual, simple clinical tests for early prognosis of two levels of favorable arm recovery at 6 months post-stroke. Patients with partial shoulder abduction were 14.6 times more likely to reach a moderate-to-mild level of residual arm impairment in the fully adjusted analysis. Partial elbow extension and finger extension were also predictors of a moderate-to-mild residual impairment in the partially adjusted analysis. Patients with full function on the selected motor tests were at least 5.5 (shoulder abduction) to 35.3 (pronation/supination) times more likely to achieve a mild level of residual motor impairment compared with those with absent function. Partial motor function was likewise associated with an almost complete arm recovery, with odds ratios ranging from at least 2.1 (elbow extension) to 18.3 (wrist dorsiflexion). Moderate motor impairment and intact sensory function were also associated with favorable outcome.

The predictive value of some of the candidate predictors (shoulder abduction, finger extension, and sensory function) has been investigated previously (see section Introduction). The current study provides additional external validation of these known predictors by exploring their ability to predict two levels of a well-defined and clinically relevant functional outcome.

Previous studies have demonstrated that simple tests predict lower levels of arm functioning corresponding to some activity capacity (≥10 or 35 points on the Action Research Arm Test) (7, 8, 19, 29) and moderate arm impairment (≥32 or 44 points on the FMA-UE) (18, 22). However, it has been reported that a much higher level of arm functioning is necessary to perform ADL on routine basis (46). Our results showed that some of our newly proposed candidate predictors (full pronation/supination, full elbow extension, partial or full wrist dorsiflexion, and full finger mass extension) provided the strongest prognosis of a mild level of residual arm impairment. Voluntary finger extension has consistently been associated with a favorable arm recovery (8, 16, 17, 22, 26, 27). It has been suggested that hand receives innervation only from the affected hemisphere (58), and the presence of voluntary finger extension reflects the preservation of some of the fibers of the corticospinal tract system in the affected hemisphere, which controls distal arm and hand muscles (8, 59, 60). Thus, the more favorable chances of achieving an almost complete arm recovery when demonstrating initially high distal arm function might be explained by the assumption of intact innervation from the affected hemisphere. Shoulder abduction and finger extension are essential components of several multivariable prognostic models (8, 16, 17, 22), and have also been reported as individual predictors (27, 61). Interestingly, in our study, active shoulder extension and finger extension were not significantly associated with an almost complete arm recovery, unless the participants were able to perform these movements fully. Thus, the ability to perform some shoulder abduction in synergistic patterns and finger mass extension might not be suitable for the prognosis of the very high level of arm functioning (FMA-UE ≥ 58) that we aimed to predict.

The large sample size (*n* = 223) and the statistical analyses adjusted for a broad range of possible confounders strengthen the internal validity of our results. Younger age, being a male and having a good lower extremity function have previously been associated with more favorable outcomes in terms of arm functioning; inconclusive evidence has been reported for type of stroke, stroke severity, cognition and time since stroke (34). Overall, the subgroups with 6-month unfavorable outcomes had a more severe stroke, more impaired arm and leg function at baseline and a longer hospital stay. Apart from the initial level of arm impairment, all these confounders were added to the fully adjusted analyses. While the fully adjusted analysis (ORf) may be over adjusted, the whole portfolio of ORu, ORp, and ORf provides a detailed image of the results. Moreover, our analyses were performed on pooled individual participant data from two independent Scandinavian stroke cohorts, strengthening the external validity of our findings.

A limitation of this study is that both the predictive value (OR) and its statistical uncertainty of a moderate-to-mild outcome (FMA-UE ≥ 32) for most motor tests were estimated as infinity. This is caused by the observed Positive Predictive Value (PPV) of 100% for these tests, and the absence of meaningful assessment of the uncertainty of the estimated predictive value. Hence, it was not possible to quantify with a single number (i.e., the OR) the predictive value of these candidate predictors. The PPV of 100% makes, however, these motor tests rather suited for prediction of moderate-to-mild residual arm impairment. These findings suggest that having some proximal (shoulder abduction, elbow extension) or distal (pronation/supination, wrist dorsiflexion, finger extension, or ability to hold a pen/cylinder) voluntary movement early after stroke are indicative for a 6-month function level that allows performance of at least simple daily tasks. Other measures for predictive values, e.g., the risk ratio (RR), could be used to avoid estimates that attain infinity. However, only the OR does not depend on the outcome distribution and therefore the external validity of other measures would be limited. For example, RRs from two different data sets cannot be readily compared.

These results are in line with findings from previous studies showing that proximal arm control (8, 29, 32, 33, 62) and sufficient sensory function (19, 29, 32) measured during the first month predict a better hand dexterity at 3 to ≥12 months after stroke.

The advantage of using the FMA-UE as outcome is that it allows prediction of improvements predominantly due to pure neurologic recovery rather than functional recovery which includes the interference of compensatory strategies (forward-bending of the upper body to compensate for impaired elbow extension in reaching tasks). Predicting functional recovery may, however, be more meaningful for patients and clinicians because it reflects the patients' ability to perform tasks that are relevant for their daily life. Thus, dichotomizing the FMA-UE according to the proposed cut-off levels of 32 (ability to perform at least basic ADL) and 58 points (probable use of the affected arm in ADL on routine basis) provides the possibility to relate initial, easy-to-measure arm function to functional ability at later stages. Finally, both the predictors and the predicted outcome are clinically relevant, easy-to-measure, and suitable for stroke patients with various degrees of arm paresis and cognitive dysfunctions, making our findings valuable for clinical practice. Our findings regarding the newly proposed predictors of an almost complete arm recovery, particularly the presence of partial wrist dorsiflexion, need to be confirmed by future studies.

The selected potential predictors are some of the single items from which the 6-month outcome was calculated. There is a natural correlation between the items at baseline and at 6 months and there is also a natural correlation between a single item score and the total FMA-UE score. Therefore, the single items at baseline will be natural candidate predictors for the 6-month outcome. One may argue that these single items are artificially good predictors for the outcome by construction. However, the 6-month outcome was dichotomized into categories that are well-established to signify specific levels of functioning connected to the ICF-activity domain. This is a different domain from the body function domain to which the predictors belong.

## Conclusion

This study showed that the presence of some shoulder abduction, elbow extension, and finger extension early post-stroke predicts a moderate-to-mild residual motor impairment at 6 months post-stroke. Importantly, we extended these findings by showing that full function on a large range of simple motor tests as well as partial function on some of these tests (i.e., wrist dorsiflexion, pronation/supination) were associated with mild residual motor impairment. Full sensory function and moderate motor impairment were also associated with a favorable outcome, but they had more modest predictive values. Thus, these easily conducted motor tests may be useful tools for early prediction of arm recovery. This knowledge can be used in the development of prognostic models feasible in regular clinical settings, inform patient selection and stratification in future trials, and guide clinicians in the selection of individualized, evidence-based training strategies for improving the affected arm functioning after stroke.

## Data Availability Statement

The dataset generated for this study are available on request to EG (emma.ghaziani@regionh.dk) and MM (margit.alt-murphy@neuro.gu.se); permissions from the Danish and Swedish Committees on Health Research Ethics are needed.

## Ethics Statement

The studies involving human participants were reviewed and approved by The Regional Ethical Review Board (225-08), Gothenburg, Sweden. The Capital Region of Denmark's Committee on Health Research Ethics (H-4-2014-012). All patients/participants provided their written informed consent to participate in the original studies.

## Author Contributions

EG: study conception and design, merging of data and preparation of the SALGOT-ESS dataset, data collection (ESS trial), statistical analyses, and manuscript preparation. CC and HC: supervision. VS: statistical analysis and manuscript preparation. SM: manuscript preparation and supervision. KS: study conception, design, and supervision. HP: study conception and design, data collection (SALGOT), and merging of data. MA: study conception and design, data collection (SALGOT), manuscript preparation, and supervision. All authors have substantially contributed to the interpretation of data, revised the manuscript critically for important intellectual content, and approved the final version.

### Conflict of Interest

The authors declare that the research was conducted in the absence of any commercial or financial relationships that could be construed as a potential conflict of interest. The design, data analysis, results, and conclusions reported in this paper are those of the authors and are independent from the funding sources.
